# Nasal delivery of thermostable and broadly neutralizing antibodies protects mice against SARS-CoV-2 infection

**DOI:** 10.1038/s41392-022-00911-5

**Published:** 2022-02-21

**Authors:** Wenhui Fan, Shanshan Sun, Ning Zhang, Yuan Zhang, Pengtao Jiao, Jian Wang, George F. Gao, Wenjun Liu, Yuhai Bi, Limin Yang

**Affiliations:** 1 CAS Key Laboratory of Pathogenic Microbiology and Immunology, Institute of Microbiology, Center for Influenza Research and Early-warning (CASCIRE), CAS-TWAS Center of Excellence for Emerging Infectious Diseases (CEEID), Chinese Academy of Sciences, Beijing, 100101 China; 2 Tianjin Speerise Challenge Biotechnology Co., Ltd, Tianjin, 300380 China; 3 University of Chinese Academy of Sciences, Beijing, 101409 China

**Keywords:** Preclinical research, Infectious diseases, Infection

**Dear Editor**,

The ongoing SARS-CoV-2 pandemic has lasted for 2 years, causing a severe global impact on social development. With widespread vaccinations, the number of new infections has dropped significantly, but with the emergence of diverse novel variants of concern (VOCs) and variants of interest (VOIs), there has been a second or third epidemic wave in many countries. Multiple variants have the potential to increase transmissibility, virulence, or evade immune responses. Among them, the Beta (B.1.351) and Omicron (B.1.1.529) VOCs have significantly decreased neutralization even in fully vaccinated individuals, while the Delta VOC (B.1.617.2) has been reported to be more infectious and has indeed become globally dominant.^1,2^ All currently licensed vaccines are designed based on the first-reported strain and exhibit deficient cross-neutralization against variants, highlighting the risk for health care and other service workers. In addition, due to the imbalance of global development, many countries still cannot obtain ample vaccines. Furthermore, studies have shown that cats and dogs are also susceptible to SARS-CoV-2, and there is currently no vaccine for pets.^3^ Given that current vaccine supplies are inadequate to meet the global demand and that it is challenging to reformulate existing vaccines to include diverse SARS-CoV-2 spike (S) antigens in a short time, so a safe, deployable, and broadly protective intervention against new evolving viruses has become particularly urgent.

Compared with vaccination, passive antibody administration can provide immediate immunity to protect susceptible persons. Convalescent plasma and monoclonal antibodies (mAbs) have been used to treat COVID-19, and multiple mAbs have been authorized for emergency use. However, antibody therapies are administered chiefly in hospitalized patients with severe pneumonia due to their high price. Furthermore, Omicron VOC caused more than 85% of mAbs to lose neutralizing activity,^2^ suggesting that mAb therapies have limitations against emerging SARS-CoV-2 variants. In recent years, antibody therapy strategies based on egg yolk antibodies (Immunoglobulin Y, IgY) have gained attention, where promising results have been reported in treating SARS-CoV, influenza virus, hantavirus, norovirus, and Ebola virus infections.^4^ Moreover, IgY antibodies have shown good thermal stability and can be stored stably in a liquid state at room temperature for 6 to 12 months. Strikingly, egg production has already been scaled up, making low-cost and large-scale IgY antibodies feasible. In the present study, we developed a broadly protective IgY antibody against SARS-CoV-2, and nasal delivery conferred significant protection against SARS-CoV-2 infection in the mice model.

A SARS-CoV-2 receptor-binding domain (RBD) trimer vaccine candidate was prepared in our previous study and showed potent protection in a nonhuman primate model.^5^ In this study, we used it to immunize laying hens. The immunization and sampling schedule is shown in Fig. 1a. To obtain broadly neutralizing antibodies (NAbs), the immunogens included wild type (WT) Wuhan-hu-1 strain RBD trimer (RBD-WT) and Beta VOC RBD trimer (RBD-Mu), which differ by three amino acids (K417T, E484K, and N501Y). Moreover, RBD-Mu covered all mutations in Alpha/Gamma VOCs and partial mutations in Omicron VOC. IgY antibodies were purified from eggs collected at different time points after vaccination, and the SARS-CoV-2 binding and neutralizing antibody levels were measured. RBD-specific ELISA titers gradually increased and peaked at 3880 (95% CI: 1797–8379) after the final immunization (Fig. 1b). Similar to the binding antibody, the live virus (WT) NAb geometric mean titers (GMTs) reached 110.7 (95% CI: 66.04–165.7) at 3 weeks after the second dose and increased to 1039 (609.3–1577) after the final immunization (Fig. 1c and Supplementary Fig. S1a).

**Fig. 1:**
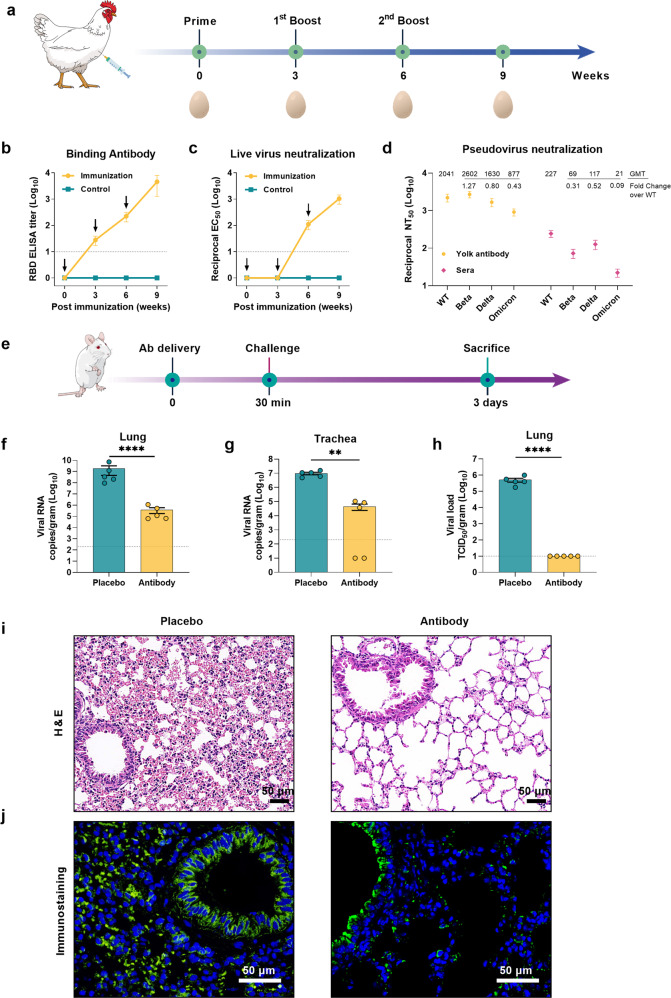
Protection of yolk antibody (IgY) against SARS-CoV-2 challenge in mice. **a** Flow chart of experimental design for preparing the SARS-CoV-2 specific yolk antibodies. Solid vertical lines indicate weeks of immunization, and egg symbols indicate sampling time points. **b**, **c** The humoral response dynamics of immunized laying hens were monitored. SARS-CoV-2 specific binding antibodies were assessed by ELISA. Neutralization activity with live SARS-CoV-2 (WT) was assessed as EC_50_ by observing the cytopathic effect (CPE). Dotted lines indicate the detection limit. See also supplementary Fig. S1a. **d** Neutralization of the Wuhan-Hu-1 (WT), Beta, Delta, and Omicron variants of concern (VOCs) by the second boost IgY antibodies were measured by a recombinant lentiviral-based pseudovirus neutralization assay. Sera from three volunteers who had received two doses of inactivated vaccines (Sinopharm) were collected and assayed simultaneously. See also supplementary Fig. S1b, c. **e** Flow chart of the experimental design for protective testing of the developed IgY antibody. Mice were intranasally (IN) transduced with 8 × 10^9^ virus particle (VP) of Ad5-hACE2 followed by nasal delivery of antibody or PBS-placebo. They were then challenged with SARS-CoV-2 and euthanized at 3 days post-infection for viral load titration and pathological evaluation. Solid lines indicate the timeline of antibodies delivery IN (black), challenge (red), and sacrifice (green). **f**, **g** Viral loads in lung and trachea tissues were measured by qRT-PCR. **h** Lung virus titers were detected by TCID_50_ assay in Vero E6 cells. **i** H&E staining of the representative lung tissue sections. Scale bars are 50 μm. See also supplementary Fig. S2. **j** Immunofluorescence staining detection of the SARS-CoV-2 S protein. Scale bars are 50 μm. Data were shown as mean ± SEM. An unpaired *t*-test was performed to test statistical significance between the group receiving the IgY antibody and the placebo group (**P < 0.01; ****P < 0.0001)

To further determine the broad-spectrum neutralization potency of the developed IgY antibody, we evaluated the neutralizing activity against the SARS-CoV-2 WT strain, Beta, Delta, and Omicron VOCs using a luminescence-based lentiviral pseudovirus neutralization assay, the sera from three volunteers vaccinated with two doses of inactivated vaccines (Sinopharm) were used as the positive control. Remarkably, the IgY antibody exhibited neutralization of the Beta, Delta, and Omicron pseudoviruses that were comparable to that of the WT pseudovirus, with GMTs of 2041 (95% CI: 640.3–6507) for WT, 2602 (95% CI: 1178–5748) for Beta VOC, 1630 (95% CI: 577.9–4596) for Delta VOC, and 877 (95% CI: 362.2–2122) for Omicron VOC (1.2-fold increase in Beta, 1.3-fold decrease in Delta, and 2.3-fold decrease in Omicron versus WT). The GMTs of volunteers’ sera were 227 (95% CI: 96.5–531.9) for WT, 69 (95% CI: 21.3–225.3) for Beta VOC, 117 (95% CI: 35.66–386.7) for Delta VOC, and 21 (95% CI: 6.2–69.1) for Omicron VOC, with an average of 3.2-fold reduction in Beta VOC, 1.9-fold reduction in Delta VOC, and 10.8-fold reduction in Omicron VOC compared to WT (Fig. 1d and Supplementary Fig. S1b, c). These results indicate that the RBD trimer bivalent (RBD-WT and RBD-Mu) vaccine candidate can elicit broadly cross-reactive NAbs. In addition, since we only tested three sera samples as control, the results can be used to evaluate the neutralization assay in this study and are not meaningful for the cross-neutralizing activity of sera.

To evaluate the protective efficacy of the IgY antibody in vivo, ten BALB/c mice were first intranasally (IN) transduced with adenovirus expressing human angiotensin-converting enzyme 2 (hACE2) as SARS-CoV-2-sensitive animal model. Five days later, the transduced mice were randomly assigned (5:5) and IN treated with either the IgY antibody (0.1 mL) or PBS-placebo. Thirty minutes later, mice were IN challenged with 5 × 10^5^ TCID_50_ SARS-CoV-2 hCoV-19/China/CAS-B001/2020 strain. All mice were euthanized at 3 days post-infection, viral loads in the lung and trachea were measured, and lung pathological changes were detected by H&E staining (Fig. 1e). The results showed that nasal delivery of IgY led to a 10^3^-fold reduction of viral RNA in the lungs, and a 10^2^-fold reduction in the trachea compared to the placebo group (10^9^ (lung) and 10^7^ (trachea) copies/gram mean titers) (Fig. 1f, g). Notably, no live virus was detected in the lungs of the IgY treated mice, indicating effective blocking of viral propagation (Fig. 1h). In addition, the lungs of the placebo group exhibited severe pathological damage, including alveolar wall thickening, inflammatory cell infiltration, and blood cell exudation. In contrast, the pathological injuries were significantly weaker in the IgY group (Fig. 1i and Supplementary Fig. S2a, b). Immunofluorescence results revealed a high amount of viral S protein in mice lungs of the placebo group, while only trace amounts of positive signals were detected in the IgY group (Fig. 1j). Taken together, the developed IgY antibody significantly reduced the virus loads and histopathological changes in the lungs of the mice challenged by a high load of SARS-CoV-2.

To confirm the feasibility of mass production, we produced the IgY antibody against SARS-CoV-2 with a pilot-scale production in GMP-grade manufacturing. Notably, IgY antibody could be produced at high quantity, with a final yield of 5.2 g purified antibody per liter of yolk. The purity and neutralizing activity were >98% and >2000 as detected by SDS-PAGE and the pseudovirus neutralization assay (Supplementary Fig. S3a). Additionally, the specificity of the IgY antibody was evaluated using immunofluorescence analysis. The results revealed that the IgY product could specifically recognize the S protein of live SARS-CoV-2 in Vero cells (Supplementary Fig. S3b). Thermal stability is vital for antibodies, so we tested a 3-month at 25 °C storage for the purified IgY antibody. The neutralizing activity and protein concentration were detected every month. Notably, the neutralizing activity remained stable throughout 3 months, and their concentration and purity did not change (Supplementary Fig. S3c, d). Together, the highly scalable production and high thermal stability are feasible to meet the demands worldwide for prevention and control of COVID-9, avoiding cold chain transportation.

In summary, we prepared a poultry-derived polyclonal IgY antibody based on SARS-CoV-2-RBD bivalent vaccine candidate, which could effectively neutralize SARS-CoV-2 WT, Beta, Delta, and Omicron VOCs. Nasal delivery of this antibody significantly protected mice against a high-dose SARS-CoV-2 challenge, indicating that passive immunoprophylaxis with antibodies via nasal spray may be an effective method for preventing post-exposure infection. In addition, the IgY antibody exhibited excellent thermal stability and high yields. Therefore, this IgY antibody promises to be supplemental pre-exposure prophylaxis to protect both individuals who have not been vaccinated and those for where immediate immunity is required as an outbreak in a crowded area. It may also help block the route of virus transmission in pets and other animals susceptible to SARS-CoV-2.

## Supplementary information

Sigtrans_Supplementary

## Data Availability

The data that support the findings of this study are openly available in Science Data Bank at 10.11922/sciencedb.01269.
