# Supplementary material for: Nasal delivery of thermostable and broadly neutralizing antibodies protects mice against SARS-CoV-2 infection
Source: Signal Transduct Target Ther. 2022 Feb 21;7:55. doi: 10.1038/s41392-022-00911-5 (PMC8860136; doi:10.1038/s41392-022-00911-5)
Supplement: Supplementary file 1 — Sigtrans_Supplementary [file 41392_2022_911_MOESM1_ESM.docx]

Supplementary Materials for

Nasal delivery of thermostable and broadly neutralizing antibodies protects mice against SARS-CoV-2 infection

Wenhui Fan^1,^**^†^**, Shanshan Sun^3,^**^†^**, Ning Zhang^1,^**^†^**, Yuan Zhang^1^, Pengtao Jiao^1^, Jian Wang^3^, George F Gao^1,2^, Wenjun Liu^1,2^, Yuhai Bi^1,2,^*, Limin Yang^1,^*

^1^CAS Key Laboratory of Pathogenic Microbiology and Immunology, Institute of Microbiology, Center for Influenza Research and Early-warning (CASCIRE), CAS-TWAS Center of Excellence for Emerging Infectious Diseases (CEEID), Chinese Academy of Sciences, Beijing 100101, China

^2^University of Chinese Academy of Sciences, Beijing 101409, China

^3^Tianjin Speerise Challenge Biotechnology Co., Ltd., Tianjin 300380, China

†These authors contributed equally to this work.

Correspondence to: L.Y. (email: [lmyang@im.ac.cn](mailto:lmyang@im.ac.cn)) or to Y.B. (email: beeyh@im.ac.cn)

**This PDF file includes:**

Materials and Methods

Figures. S1 to S3

Materials and Methods

Ethical statement

All animal experiments were approved by the Research Ethics Committees of Institute of Microbiology, Chinese Academy of Sciences (CAS) and performed according to the Animal Experimentation Guidelines (approve no. SQIMCAS2021157). All applicable institutional and/or national guidelines for the care and use of animals were followed. Three serum samples collected from three volunteers who had received two full doses of inactivated vaccines (Sinopharm) were used as positive control in this study.

Animals

Laying hens were purchased from Tianjin Speerise Challenge Biotechnology Co., Ltd. Female BALB/c mice (6-8-weeks old) were purchased from Beijing Vital River Laboratory Animal Technology Co., Ltd. All animals were kept in sterile, autoclaved cages and provided with enough food and water.

Cell lines and virus

African green monkey kidney epithelial cell line (Vero-E6) (ATCC, no. 1586) was used for SARS-CoV-2 propagation and NAb determination. The SARS-CoV-2 strain, hCoV-19/China/CAS-B001/2020 (CAS-B001) (National Microbiology Data Center NMDCN0000102-3, GISAID databases EPI_ISL_514256-7), used in this study was isolated and identified by Dr. Yuhai Bi's team, and was stored in the BSL-3 laboratory of Institute of Microbiology, Chinese Academy of Sciences.

Immunization

5-month-old laying hens (n=5) were immunized three times (weeks 0, 3, 6) by intramuscular injection route in the chest with a total volume of 1 mL of vaccine formula containing 20 μg of RBD-trimer (10 μg RBD-WT and 10 μg RBD-Mu) admixed with white oil adjuvant (Sinopec Hangzhou Refinery) and placebo (PBS)-treated laying hens (n=5) were used as negative control. To assess immune response after immunization, eggs were collected at weeks 0, 3, 6, 9.

Purification of yolk antibody

Yolk antibodies (IgY) were extracted from the egg yolks using the water dilution method according to our previous research ^3^. Yolks without membrane were separated and diluted at 1:8 with cold deionized water. Dilution was stirred and acidified to pH 5.0, keeping stability for 10 h then centrifuged at 10 000 × g for 15 min, and the supernatant was collected, 1% (by volume) of the n-octanoic acid was then slowly added to the supernatant and was centrifuged in the same way. Finally, the IgY antibody was further purified by gel filtration (Superdex 200, GE Life), eluted in PBS (pH 7.2) buffer, and concentrated by ultrafiltration to approximately 20 mg/ml using 100-kDa cut-off membranes (Millipore).

Challenge

Ten BALB/c mice were intranasally (IN) transduced with 8×10^9^ virus particle (VP) of recombinant adenovirus serotype 5 (Ad5) expressing human angiotensin-converting enzyme 2 (hACE2). These SARS-CoV-2-sensitive mice were anesthetized with isoflurane and delivered 0.1 mL yolk antibody or PBS-placebo via IN route at day 5. Thirty minutes after antibody delivery, the mice were IN challenged with a dose of 5 × 10^5^ TCID_50_ SARS-CoV-2 strain CAS-B001 in a volume of 50 μL. Mice were monitored daily after the challenge and euthanized at 3 dpi. Lung and trachea tissues were collected. All experiments with live viruses and animals were performed in BSL-3 and complied with the instruction of the institutional biosafety manual.

Viral load measurement

Viral RNA was extracted from homogenized lung and trachea tissues using a QIAamp® Viral RNA Mini Kit (QIAGEN, Germany). Reverse transcription and TaqMan quantitative PCR were performed using a one-step PrimeScript^TM^ RT-PCR Kit (TaKaRa) according to the instructions on an ABI 7500 Real-Time PCR System. Primers and probes were specific for SARS-CoV-2 N gene, as follows: F: 5'- GGGGAACTTCTCCTGCTAGAAT -3', R: 5'- CAGACATTTTGCTCTCAAGCTG -3'; probe: 5'-FAM-TTGCTGCTGCTTGACAGATT-TAMRA -3'). The 25 μL reaction mixtures were set up with 8 μL of viral RNA. The cycling conditions were as follows: 42°C for 5 min, 95°C for 10 s, and 40 cycles of 95°C for 5 s and 60°C for 31 s. Serial dilutions of SARS-CoV-2 RNA reference material (National Institute of Metrology, China) were used for a standard curve of qRT-PCR. The copy number of each sample was calculated with the standard curve. Viral loads were expressed as viral copies/g. The limit of detection was 200 copies/g.

ELISA

A SARS-CoV-2-RBD-specific ELISA was employed to determine endpoint binding antibody titers of yolk antibodies harvested from immunized hens. Endpoint titers were defined as the reciprocal highest antibodies dilution that yielded an OD_450_ value > 2-fold over that of the negative values. Briefly, 96-well ELISA plates (Corning, USA) were coated with 10 μg/ml recombinant RBD protein (1μg/mL, Sino Biological) in carbonate-bicarbonate buffer (pH 9.6) at 4°C overnight and blocked with 4% bovine serum albumin in PBS containing 0.05% Tween-20 (pH 7.4) at 37°C for 1 h. Starting with 20-fold dilution, yolk antibodies were then serial 2-fold diluted and added to the wells following by incubation at 37°C for 1 h. After 3 washes with PBST, the plates were incubated with an HRP-conjugated goat anti-chicken IgY antibody (bs-0310G-HRP, Bioss) working solution (5000-fold dilution) at 37°C for 30 min. After 3 washes with PBST, the assay was developed by adding 100 μL of 3,3’,5’,5-tetramethylbenzidine (TMB) to each well and terminated by adding 50 µl of 2 M H_2_SO_4_ for 10 min. The value of each well was measured at OD_450 nm_ by a microplate reader using Softmax Pro 6.0 software (Molecular Devices, CA, USA).

Indirect immunofluorescence assay (IFA)

Vero E6 cells were seeded in 24-well plates with cover slides at a concentration of 2 × 10^5^ cells/mL and incubated for 12 h at 37 °C under 5% CO_2_ in DMEM medium supplemented with 10% fetal bovine serum (FBS; Thermo Fisher Scientific). The cells were infected with SARS-CoV-2 (MOI = 1) and were analyzed by IFA at 48 h post-infection. In brief, the infected cells were fixed with 4% paraformaldehyde at 4 °C for 72h, and the fixed cells were blocked with blocking buffer (PBS with 0.5% Triton X-100 and 4% BSA) for 2 h at 37 °C, then the cells were incubated with the purified IgY antibody for 1h at 37 °C, followed by three times washes with PBST (PBS with 0.5% Triton X-100) and incubation with TRITC-conjugated goat anti-chicken IgY(H+L) antibody (200-fold diluted in blocking buffer) for 1 h at 37 °C. After washing 3 times with PBST, DAPI (5000-fold dilution) was used to stain the nuclei for 20 min at room temperature. Then the cells were imaged using a confocal laser scanning fluorescence microscope (Leica SP8).

Neutralization assays

1. Live SARS-CoV-2 neutralization assay

The live SARS-CoV-2 neutralization assay was conducted in the BSL-3 laboratory based on the neutralizing activities against SARS-CoV-2 CAS-B001 (WT) infection in Vero E6 cells. In brief, Vero E6 cells were seeded in 96-well plates at a concentration of 2 × 10^5^ cells/mL and incubated for 12 h at 37 °C with 5% CO_2_ in DMEM medium supplemented with 10% fetal bovine serum. The purified yolk IgY antibodies obtained from immunized eggs were heat-inactivated at 56°C for 30 min and serially diluted with dilution medium at a starting dilution of 1:10 and then diluted 2-fold up to 1:2560. An equal volume of challenge virus solution with a concentration of 2000 TCID_50_/mL was added to the antibody dilutions, followed by incubation at 37°C for 1h. The antibody-virus mixture (100 μL /well) was then added to the Vero E6 cells and the plates were incubated for 3 days at 37°C under 5% CO_2_. Cytopathic effect (CPE) of Vero E6 cells in each well was recorded under microscopes and the neutralizing titer (50% effective concentration, EC_50_) was determined by Reed-Muench method using GraphPad Prism 8.0 software (GraphPad Software, CA, USA).

1. Pseudovirus neutralization assay

For pseudovirus production, the full-length S protein plasmid pCAGGS-S-Wuhan-Hu-1(GenBank: MN908947.3), pCAGGS-S-B.1.351 (GISAID EPI_ISL_1250476), pCAGGS-S-B.1.617.2 (GISAID EPI_ISL_2029113), or pCAGGS-S-B.1.1.529 (GISAID: EPI_ISL_6752027) were transfected together with the packaging construct pPAX2 and luciferase reporter plasmid pLenti-GFP into 293T cells at a ratio of 1:1:1. The pseudovirus supernatant was harvested at 48-72h and the TCID_50_ was determined based on the relative luciferase activity in the pseudovirus infected hACE2/293T cell lysates. hACE2/293T cells were seeded in 96-well plates with a concentration of 2 × 10^5^ cells/mL and incubated for 12 h at 37 °C with 5% CO_2_ in DMEM medium supplemented with 10% fetal bovine serum. Yolk IgY antibody or serum samples were heat-inactivated at 56°C for 30 min and serially diluted with dilution medium at a starting dilution of 1:20 and then diluted 2-fold up to 1:10240. An equal volume of SARS-CoV-2 pseudovirus (2000 TCID_50_/mL) was added, followed by a 1 h incubation at 37°C. Then 100 μL of the mixture was added to the hACE2/293T cells and the plates were incubated for 24 h at 37°C under 5% CO_2_ and 100 μL/well fresh media was added. At 72h after infection, cells were lysed using passive lysis buffer and the luciferase activities were generated with Luciferase substrate (Promega) and read by a multimode plate reader. Neutralization was calculated as the percent change in relative luminescence units (RLUs) in the presence of pre-immune antibody versus that of RLUs in the presence of immune antibody [(Pre-immune RLUs − Immune RLUs)/(Pre-immune RLUs)]×100. The NAb titers were determined using Reed-Muench method at the antibody dilution with 50% neutralization (NT_50_) using GraphPad Prism 8.0 software (GraphPad Software, CA, USA).

Histopathological analysis

For histological examination lung tissues were fixed overnight in 4% paraformaldehyde (PFA), dehydrated, and embedded in paraffin. Subsequently, 5-μm-thick tissue sections were stained with hematoxylin and eosin (H&E) staining. For quantitative assessment of SARS-CoV-2 infection-associated pathological change in the lung, each H&E-stained section was scored using a panoramic scan image, and the score was evaluated by one independent observer blinded to the experimental conditions according to International Harmonization of Nomenclature and Diagnostic Criteria (INHAND) (<https://www.toxpath.org/inhand.asp>) and described as follow. The pathological changes in six sections (alveolar wall, alveolar space, epithelial cells of bronchi, bronchiole, vascular endothelial cells, vessel) are respectively graded as foul levels: 0, no lesion; 1, slight lesions; 2, moderate lesions and account for 1/3; 3, severe lesions and account for 1/2; 4, very severe lesions and account for 3/4.

Immunostaining analysis

5-μm-thick paraffin lung sections were deparaffinized with xylene and dehydrated with ethanol. The slices were placed in a humidified chamber filled with citrate buffer (pH6.0) and treated with microwave for antigenic repair, followed by cooling at room temperature and washing with PBS, and then blocked for 30 min with sterile PBST containing 4% BSA at room temperature. Afterward, slices were incubated with primary antibody (rabbit polyclonal antibodies against SARS-CoV-2-S1 at 0.1 µg/ml; SinoBiological, Beijing, China) for 2 h in a humidified chamber at 37℃ and washed with PBS for 3 times. FICT-conjugated mouse anti-rabbit IgG (Bioss, Beijing, China) was dropped onto the slices and incubated for 1 h at 37℃, followed by washing 3 times with PBS. The nuclei were stained with DAPI for 10 min, and then the endogenous peroxidases were quenched with hydrogen peroxide for 10 min. After extensive washing with distilled water, images were acquired and analyzed using the confocal laser scanning fluorescence microscope (Leica SP8).

Statistical analysis

All statistical analyses were performed using GraphPad Prism 8.0 software (GraphPad Software, CA, USA). Statistical significance among different groups was determined by Student's *t*-test, and differences between the experimental groups were considered statistically significant at the *P*<0.05 level.

Figure. S1.


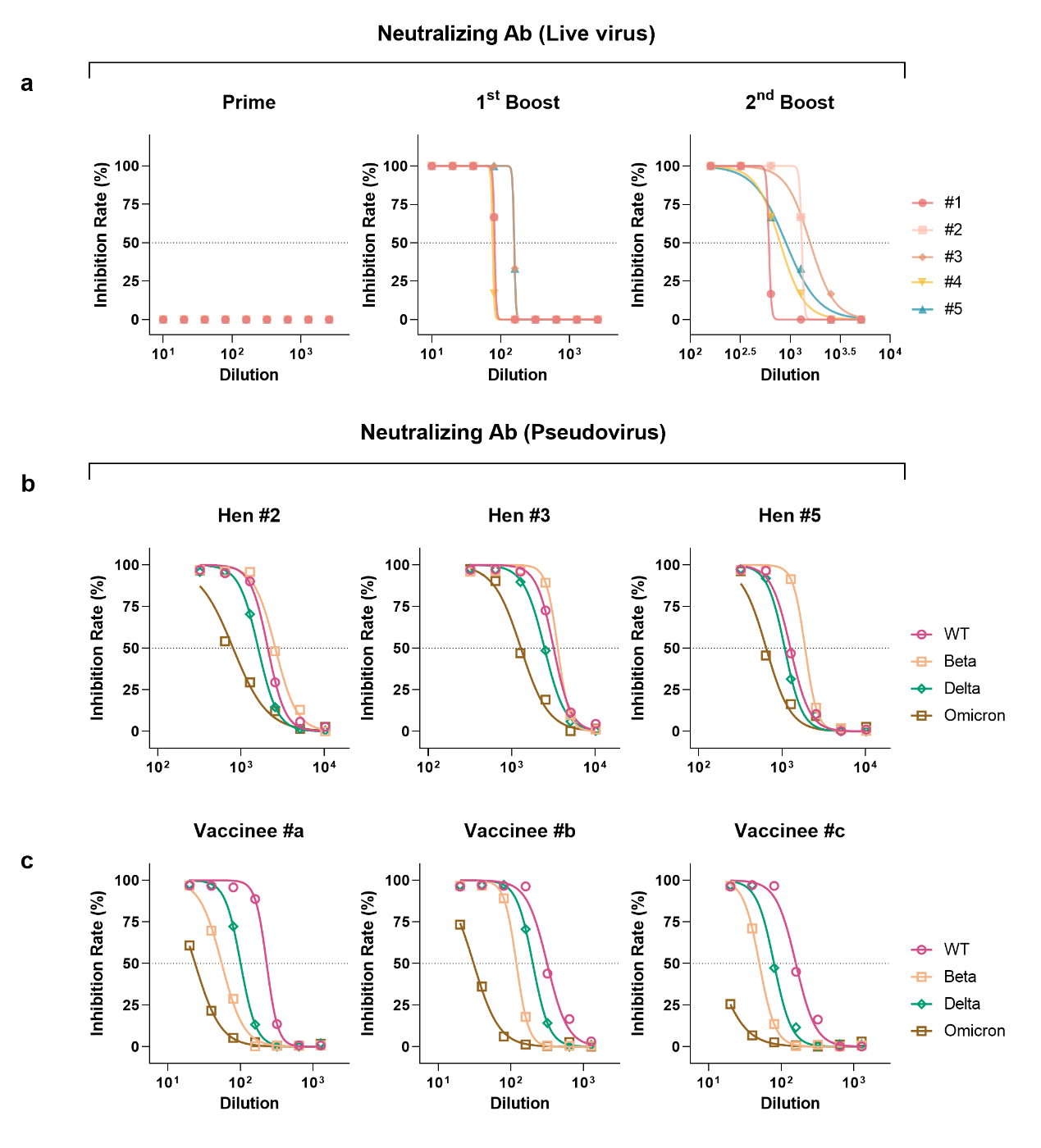


**Supplementary Fig. S1. Neutralization Assays of the Yolk IgY Antibody against SARS-CoV-2.**

1. Eggs from the immunized laying hens were collected after each immunization, and the purified yolk IgY antibodies were used for testing NAb titers with live SARS-CoV-2 (CAS-B001 strain).

**b** and **c** The yolk IgY antibodies produced at three weeks post the final immunization and the serum samples from three volunteers with two full doses of inactivated vaccines were used for testing NAb titers by LVV-PsN assay. Serial dilutions of samples were pre-incubated with pseudoviruses bearing the SARS-CoV-2-S protein from WT, Beta VOC, Delta VOC, and Omicron VOC, and the percentage inhibition of cell infection was measured. Nonlinear regression was performed using a log (inhibitor) versus normalized response curve, and a variable slope model (R^2^> 0.95 for all curves). The EC_50_ (live virus neutralization) and NT_50_ (pseudovirus neutralization) were calculated by the Reed-Muench method.

Figure. S2.


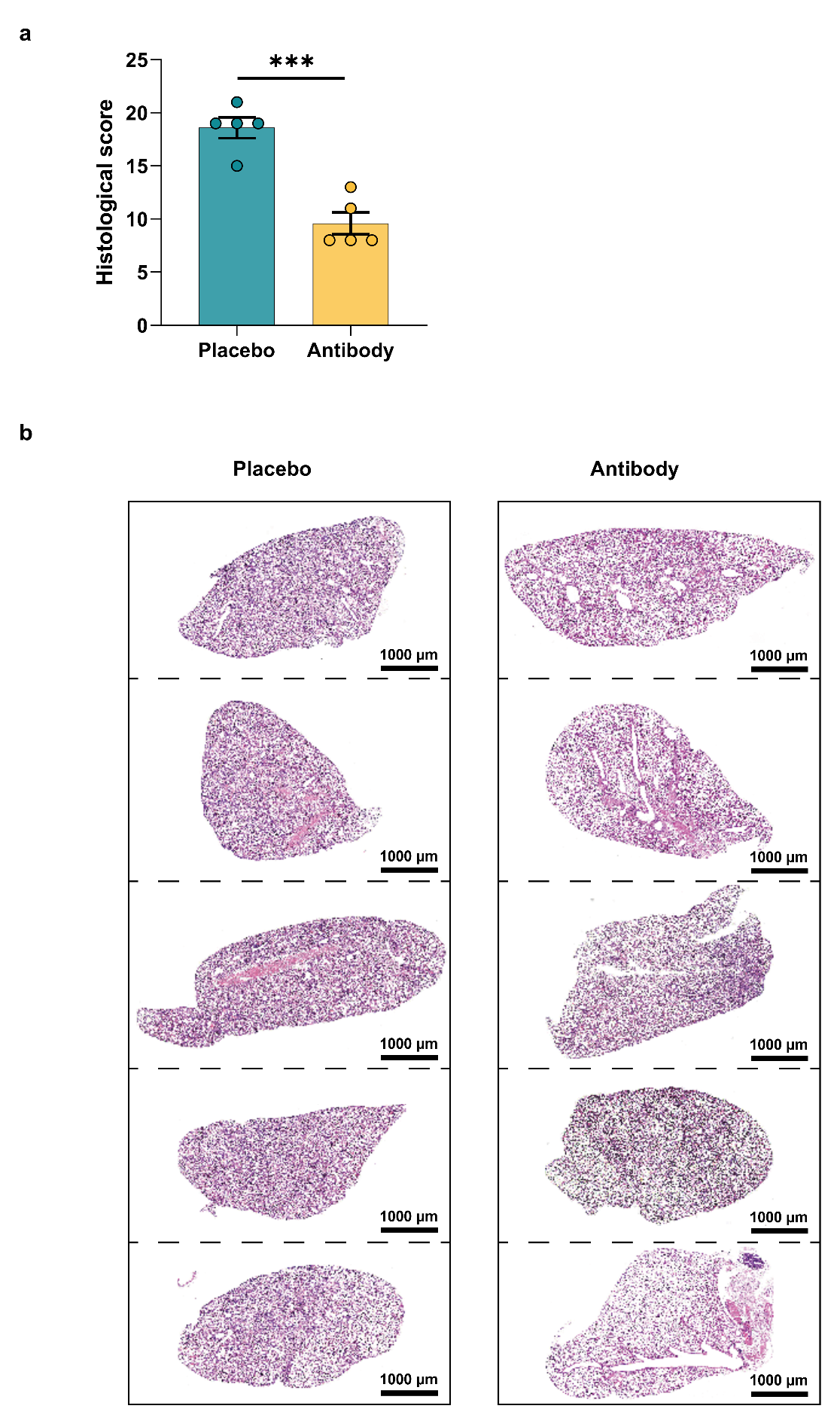


**Supplementary Fig. S2. Histological Images and Quantification Scores in Lungs of the Challenged Mice.**

1. Lung tissue sections from all mice were scored for the amount of inflammation and tissue damage among alveolar wall, alveolar space, epithelial cells of bronchi, bronchiole, vascular endothelial cells, vessel on separate scales from 0 to 4. Data are shown as mean ± SEM. An unpaired *t*-test was performed to indicate statistical significance between the antibody treat group and the placebo group (****P* < 0.001).
2. H&E staining of the lung tissue sections.

Figure. S3.


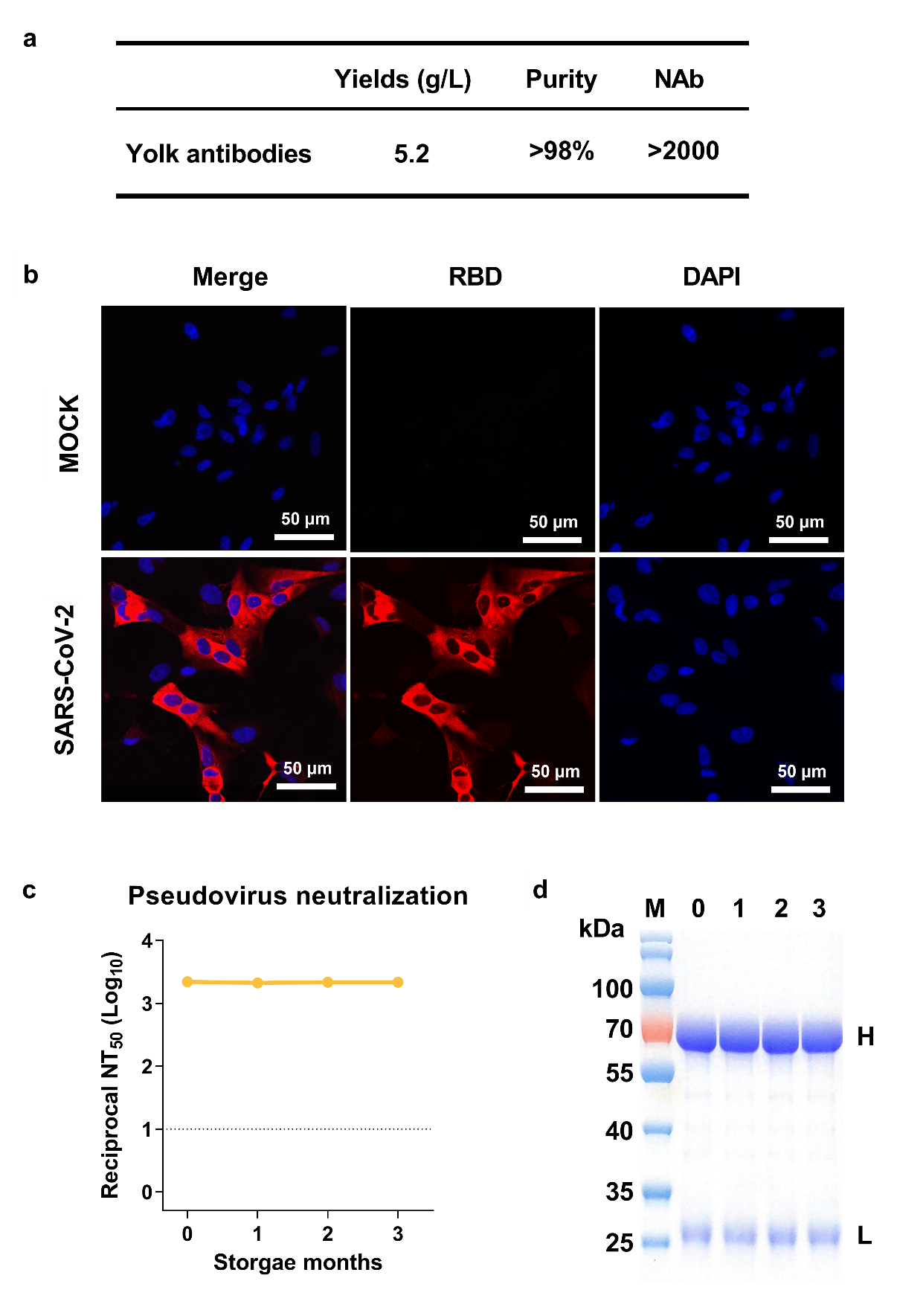


**Supplementary Fig. S3. Characterization of Yolk IgY Antibody against SARS-CoV-2**

1. SARS-CoV-2-RBD yolk antibodies were purified in GMP grade manufacturing. The yield, purity and neutralization titer for the produced antibodies are shown.
2. Immunofluorescence analysis of live SARS-CoV-2 tested with yolk IgYantibody. Vero E6 cell-infected SARS-CoV-2 were detected with yolk IgY antibody at 24 h post-infection. Red indicates viral infected cells and blue represents cell nuclei.

**c** and **d** The purified yolk IgY antibody solution was stored at 25 °C for three months. NAb titers were tested each month, and stability was analyzed by SDS-PAGE. M: Marker; lane 0-3 represent the storage times (month) for the IgY antibody.
